# Post‐Pleistocene differentiation in a Central Interior Highlands endemic salamander

**DOI:** 10.1002/ece3.5619

**Published:** 2019-08-27

**Authors:** Jacob J. Burkhart, Emily E. Puckett, Chelsey J. Beringer, Christine N. Sholy, Raymond D. Semlitsch, Lori S. Eggert

**Affiliations:** ^1^ Division of Biological Sciences University of Missouri Columbia MO USA; ^2^ Department of Biological Sciences University of Memphis Memphis TN USA

**Keywords:** *Ambystoma annulatum*, Ambystomatidae, amphibians, biogeography, Caudata, Ouachita Mountains, Ozark Highlands, Urodela

## Abstract

**Aim:**

For many endemic species with limited dispersal capacities, the relationship between landscape changes and species distributions is still unclear. We characterized the population structure of the endemic ringed salamander (*Ambystoma annulatum*) across its distribution in the Central Interior Highlands (CIH) of North America, an area of high species endemism, to infer the ecological and evolutionary history of the species.

**Methods:**

We sampled 498 individuals across the species distribution and characterized the population genetic structure using nuclear microsatellite and mitochondrial DNA (mtDNA) markers.

**Results:**

*Ambystoma annulatum* exist in two strongly supported nuclear genetic clusters across the CIH that correspond to a northern cluster that includes the Missouri Ozark populations and a southern cluster that includes the Arkansas and Oklahoma Ozarks and the Ouachita Mountains. Our demographic models estimated that these populations diverged approximately 2,700 years ago. Pairwise estimates of genetic differentiation at microsatellite and mtDNA markers indicated limited contemporary gene flow and suggest that genetic differentiation was primarily influenced by changes in the post‐Pleistocene landscape of the CIH.

**Main Conclusions:**

Both the geologic history and post‐European settlement history of the CIH have influenced the population genetic structure of *A. annulatum*. The low mtDNA diversity suggests a retraction into and expansion out of refugial areas in the south‐central Ozarks, during temperature fluctuations of the Pleistocene and Holocene epochs. Similarly, the estimated divergence time for the two nuclear clusters corresponds to changes in the post‐Pleistocene landscape. More recently, decreased *A. annulatum* gene flow may be a result of increased habitat fragmentation and alteration post‐European settlement.

## Graphical abstract

We characterized the population structure of the endemic ringed salamander (*Ambystoma annulatum*) across its distribution in the Central Interior Highlands of North America, an area of high species endemism, to infer the ecological and evolutionary history of the species. Both the geologic history and post‐European settlement history of the CIH have influenced the population genetic structure of *A. annulatum*.

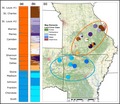

## 1 INTRODUCTION

Species distributions are governed by factors that operate across ecological and evolutionary time frames, such as changes in temperature and precipitation patterns, shifts in species interactions, and demographic stochasticity (Brown, Stevens, & Kaufman, 1996; Hewitt, 1996; Roy, Valentine, Jablonski, & Kidwell, 1996). Integrating information about the structure and dynamics of species' contemporary geographic ranges with the geologic history of a landscape allows for a better understanding of the ecology and evolutionary history of a species. Processes acting over evolutionary time frames (e.g., shifts in climate associated with glaciation events, speciation events associated with changing landscape dynamics during geological formations, shifts in paleodrainages) have lasting effects on the structure of species ranges (Hewitt, 1996). For instance, contraction of species distributions into refugial areas during the glaciation cycles of the Pleistocene (2.6–0.012 Ma) led to high rates of genetic differentiation within species (Hewitt, 2000; Lee‐Yaw, Irwin, & Green, 2008; Puckett, Etter, Johnson, & Eggert, 2015; Zamudio & Savage, 2003) and in some cases allopatric speciation (Avise & Walker, 1998; Knowles, 2000; Maddison & McMahon, 2000; Smith & Farrell, 2005).

The Central Interior Highlands (CIH) ecoregion of North America, which includes the Ozark Highlands and Ouachita Mountains of Missouri, Arkansas, and Oklahoma, has experienced shifts in species assemblages over ecological and evolutionary time frames, resulting in high levels of endemism (Soltis, Morris, McLachlan, Manos, & Soltis, 2006). This ecoregion was once part of a larger highland region that included the Appalachian Mountains to the east (Mayden, 1985). Geologic evidence suggests that separation of the highlands into Eastern and Central regions occurred during the Cretaceous to mid‐Miocene (65–12 Ma; Cushing, Boswell, & Hosman, 1964; Galloway, Whiteaker, & Ganey‐Curry, 2011). Although the Ozarks and Ouachitas date to the Ouachita orogeny of the Pennsylvanian epoch (318–299 Ma), they were formed by distinct geological processes and have been disjunct since their formation (Thornbury, 1965). The Ozarks are a northeast to southwest oriented limestone uplift with large amounts of karst habitat whereas the Ouachitas are an east–west‐oriented range of folded mountains (Thornbury, 1965). During the Pleistocene, the CIH were further bisected by the development of the Arkansas River system between the Ozarks and Ouachitas (Mayden, 1985; Wiley & Mayden, 1985). As a result of its complex geologic history, the CIH have served as a refugium for species during glacial cycles as well as the center of origin for many other species, notably fishes (Mayden, 1985; Near & Keck, 2005). During the Pleistocene glacial cycles, temperate habitats shifted south to the Gulf Coast and the CIH was covered with boreal flora (Soltis et al., 2006). At present, this ecoregion is composed of temperate broadleaf forest and mixed broadleaf forest (Flader, 2004) that contains more than 200 endemic species, of which over 160 are Ozark endemics and 48 are Ouachita endemics (Buhay & Crandall, 2005; Crandall, 1998; Mayden, 1988; Ouachita Ecoregional Assessment Team, 2003; Ozarks Ecoregional Assessment Team, 2003).

Phylogeographic studies of CIH species have found little concordance in the observed patterns of intraspecific genetic structure (Hardy, Grady, & Routman, 2002). For some taxa, the Arkansas River Valley is a barrier to movement, creating distinct Ozark Highland and Ouachita Mountain groups (Grady, Cashner, & Rogers, 1990; Herman & Bouzat, 2016; Puckett et al., 2014). In plethodontid salamanders (Herman & Bouzat, 2016; Martin, Shepard, Steffen, Phillips, & Bonett, 2016) and freshwater fishes (Grady et al., 1990; Hardy et al., 2002), genetic differentiation was found to be lower between the Eastern Highlands and the CIH than observed between the Ozarks and Ouachitas; thus, the two CIH regions were likely colonized independently and have experienced low rates of gene flow. However, other taxa, especially aquatic taxa, cluster more strongly based upon watersheds within the CIH than geologic formation, creating phylogeographic breaks between the northern Ozark Highlands of Missouri, where rivers drain into the Missouri and Mississippi Rivers, and southern populations in the Arkansas and Oklahoma Ozarks, where rivers drain into the Arkansas River (Hardy et al., 2002; Hutchison & Templeton, 1999; Routman, Wu, & Templeton, 1994).

Although aquatic and terrestrial species exhibit similar patterns of gene flow across broad scales that largely correspond to major drainages, there is less concordance at finer scales (i.e., subdrainage basins within a watershed). For aquatic organisms, gene flow is higher within subdrainage basins than among subdrainage basins as movement is constrained within aquatic ecosystems (Crowhurst et al., 2011; Hardy et al., 2002). Movements of terrestrial species, however, are less constrained, and greater genetic differentiation has been observed along geological formations (Herman & Bouzat, 2016; Martin et al., 2016) and state boundaries (Hutchison & Templeton, 1999), even within reintroduced species (Puckett et al., 2014). The observation of biogeographic breaks at political boundaries not designated based on the geology of the region (such as major rivers) suggests that differences in historic land use may have affected upland terrestrial habitats. After European settlement, the CIH experienced extensive habitat alteration (Cutter & Guyette, 1994; Nigh, 2004) that negatively influenced many endemic species through decreased dispersal among isolated glade habitats (Hutchison & Templeton, 1999) and increased siltification and eutrophication of streams leading to decreased reproductive success (Wheeler, Prosen, Mathis, & Wilkinson, 2003).

**Figure 1:**
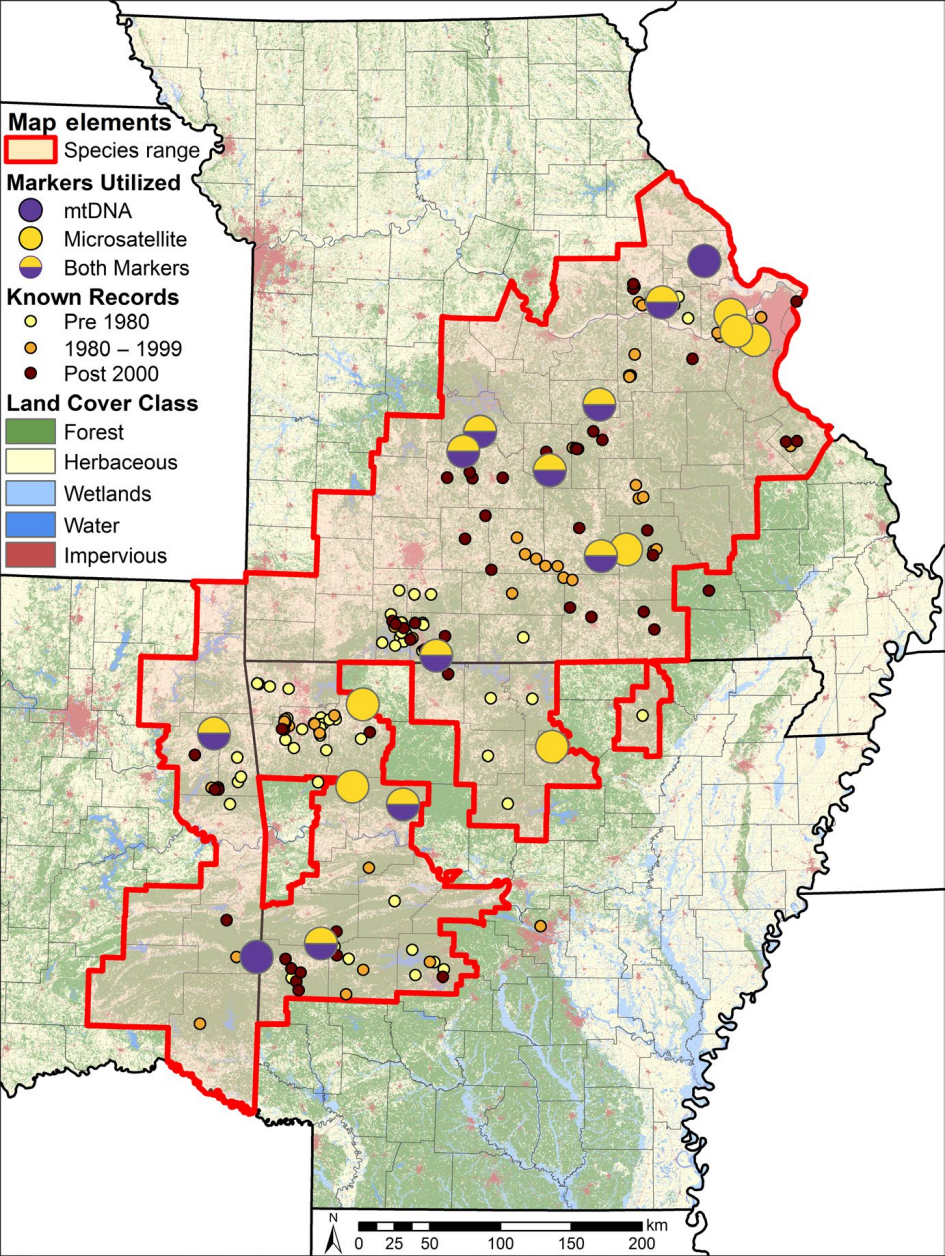
Known occurrence locations and sampling sites for *Ambystoma annulatum* within the International Union for the Conservation of Nature (IUCN) distribution overlaid on a simplified 2011 Land Use, Land Cover layer. “Forest” habitats include broadleaf, mixed, and coniferous forests, and other shrub/scrub land use classes. “Herbaceous” habitats include agricultural fields, prairie, and other herbaceous habitats. Sampling sites are indicated by the large circles

One such CIH endemic species is the ringed salamander (*Ambystoma annulatum* Cope, 1886) which is a species of conservation concern across its distribution (Figure 1; Petranka, 1998; Semlitsch, Anderson, Osbourn, & Ousterhout, 2014). *Ambystoma annulatum* has a complex life cycle in which adults make annual breeding migrations to fish‐free, semi‐permanent ponds during rainfall events between early September and late November. Females oviposit egg masses within ponds, larvae overwinter, and juveniles metamorphose the following April to June and disperse into terrestrial habitats (Hocking et al., 2008; Petranka, 1998). During the juvenile and adult life stages, *A. annulatum* are fossorial, residing in small mammal burrows or decaying root tunnels within temperate forest habitat (Osbourn, Connette, & Semlitsch, 2014). Upon reaching sexual maturity, 70%–91% of juveniles return to their natal ponds to breed (Gamble, McGarigal, & Compton, 2007; Trenham, Koenig, & Shaffer, 2001).

Previous genetic studies indicate that *A. annulatum* have been influenced by processes across evolutionary and ecological time frames. Using mitochondrial DNA (mtDNA) restriction fragment length polymorphisms (RFLPs) on samples collected across the Missouri portion of the species range, Phillips, Suau, and Templeton (2000) found three haplotypes with a south to north gradient of decreasing genetic diversity. The most common haplotype was found across all sampling sites, whereas the other two haplotypes were only found in southern Missouri, toward the center of the species distribution. Their results suggested that the fluctuating margins of temperate forest and prairie habitats during the Hypsithermal intervals of the Holocene (12 ka – present) resulted in repeated expansions and contractions of the northern periphery of the range and thus lower genetic diversity in the northern *A. annulatum* populations. More recent studies using microsatellite loci observed two genetic clusters over an approximately 7,000 ha landscape (Burkhart et al., 2017; Peterman et al., 2015), which indicates the potential for dispersal limitation over more ecological time frames.

Herein, we employ nuclear microsatellite genotypes and mitochondrial DNA sequences to estimate the genetic diversity and differentiation across ecological and evolutionary time scales for *A. annulatum*. We increase the breadth of geographic sampling to encompass the entire distribution of *A. annulatum* which allowed us to characterize the patterns of genetic population structure and diversity of *A. annulatum* across its distribution, infer phylogeographic breaks throughout the range, and infer the species demographic history. We hypothesized that (a) overall genetic diversity across the range will be low as a result of the range contraction during the Pleistocene; (b) the southern Ozark Highlands or Ouachita Mountains will have higher genetic diversity as would be expected given a northern recolonization of the CIH following the Pleistocene glacial cycles; and (c) gene flow will be limited as a result of land use changes post‐European settlement (e.g., timber harvest, fire suppression, reforestation) given that *A. annulatum* is a forest‐dependent species and is sensitive to landscape perturbations.

## 2 MATERIALS AND METHODS

### 2.1 Study area and sample collection

We surveyed breeding ponds where *A. annulatum* are known or presumed to occur across the Ozark Highlands and Ouachita Mountains in Missouri, Arkansas, and Oklahoma (Figure 1). These regions are dominated by temperate broadleaf and mixed forests (Ouachita Ecoregional Assessment Team, 2003; Ozarks Ecoregional Assessment Team, 2003). To balance representing the maximal *A. annulatum* genetic diversity across the range and the number of sampling sites, we sampled up to 20 individuals per pond from up to two ponds per county that were separated by >1.5 km. In total, we collected 498 *A. annulatum* tissue samples from 19 sampling sites across the Ozark Highlands and Ouachita Mountains during fall 2014, spring 2015, and fall 2015, representing two breeding cohorts (Figure 1). Due to low sample sizes at two of the sites, only 17 of the 19 sampling sites were utilized for microsatellite data analysis. Our tissue samples consisted of one whole embryo per egg mass, an entire hatchling, or a 0.5 cm tail clip of larval or adult *A. annulatum*. When collecting tissue samples from hatchlings and larvae, we systematically sampled from the entire perimeter of the pond to minimize the probability of sampling full siblings. Although we collected samples from different breeding cohorts, previous work in *Ambystoma* spp. salamanders shows that effective population size and genetic diversity does not significantly differ among life stages (Peterman, Brocato, Semlitsch, & Eggert, 2016) and genetic inference is largely concordant among breeding seasons across the same landscape (Burkhart et al., 2017; Peterman et al., 2015). All field and laboratory protocols were approved under the University of Missouri Animal Care and Use Committee (7403 and 8402), and collection permits were granted by the Arkansas Game and Fish Commission (032620141), the Mark Twain National Forest, Missouri Department of Conservation (15203 and 15988), Oklahoma Department of Wildlife (6091), the Ozark National Forest, and the Ouachita National Forest.

### 2.2 Molecular analysis

*Microsatellite DNA*—We extracted DNA from tissue samples using InstaGene (Bio‐Rad) following the protocols outlined by Peterman, Connette, Spatola, Eggert, and Semlitsch (2012). We genotyped individuals from 17 of the 19 sampling sites at 14 microsatellite loci (Peterman et al., 2012, 2013). We did not include the Lincoln and Le Flore sites because they had insufficient sample sizes for microsatellite analyses (*n* = 6 and *n* = 2, respectively). Forward primers were fluorescently labeled and amplified in two multiplexed polymerase chain reactions (PCR; Peterman et al., 2013) following the protocol outlined in Peterman et al. (2012). We sized the amplified products on an ABI 3730xl DNA Analyzer (Applied Biosystems) using Liz 600 size standard at the University of Missouri DNA Core Facility and scored genotypes using GeneMarkerv.1.97 (Softgenetics). Every multiplex plate included a negative control to detect contamination and a positive control to standardize scoring among plates. We genotyped 10% of samples twice to ensure quality of genotype calls.

We tested for the presence of full siblings using colony v2.0.5.9 (Jones & Wang, 2010). Within colony, we set male and female mating to polygamous without inbreeding and ran a long run with full likelihood, high precision, and no sibship prior. We tested for significant linkage disequilibrium (LD) and deviations from Hardy–Weinberg Equilibrium (HWE) with genepop on the web (Raymond & Rousset, 1995; Rousset, 2008). All tests were conducted with 1,000 dememorization steps and 100 batches with 1,000 iterations per batch, and we assessed significance using a Bonferroni correction for the number of comparisons (Rice, 1989). We tested for the presence of null alleles using the “PopGenReport” package (Adamack & Gruber, 2014) for R v3.3.3 (R Core Team, 2018). We used hp‐rare (Kalinowski, 2005) to calculate rarefied allelic richness (*A*_R_) and GenoDive v2.0 (Meirmans & Van Tienderen, 2004) to calculate observed and expected heterozygosity (*H*_E_ and *H*_O_), pairwise fixation index values (*F*_ST_; using 9,999 permutations), and inbreeding coefficients (*F*_IS_). We tested for isolation by distance (IBD) using the “vegan” package (Oksanen et al., 2016) for R, and we tested for significant differences in our genetic diversity estimates between genetic clusters and core versus periphery populations using Kruskal–Wallis tests in R. Finally, we tested for the presence of a recent genetic bottleneck using the program Bottleneck v1.2.02 (Cornuet & Luikart, 1996) using the Wilcoxon sign rank test for heterozygote excess under 10,000 iterations of a two‐phase mutation model (TPM) with 95% single‐step mutations and 5% multistep mutations and a variance among multiple steps of approximately 12 (Piry, Luikart, & Cornuet, 1999).

To assess population genetic clustering, we used the Bayesian clustering algorithms implemented in the program structure v2.3 (Pritchard, Stephens, & Donnelly, 2000). In structure, we used 2.5 × 10^5^ burn‐in steps followed by 5.0 × 10^5^ MCMC iterations for ten replicates of each *K* = 1–20 under an admixture model with correlated allele frequencies and no location prior. We estimated the most likely number of genetic clusters inferred by structure using the Δ*K* algorithm of Evanno, Regnaut, and Goudet (2005) as implemented in structure harvester v0.6.94 (Earl & vonHoldt, 2012), averaged the model outputs for the ten technical replicates using clumpp v1.2 (Jakobsson & Rosenberg, 2007), and visualized the results using distruct v1.1 (Rosenberg, 2004). We then tested for hierarchical genetic substructure within each putative cluster in a separate analysis for ten replicates of *K* = 1–15 following the same protocols as the initial analyses. We conducted a hierarchical analysis of molecular variance (AMOVA) in arlequin v.3.5 (Excoffier & Lischer, 2010) with data grouped into STRUCTURE clusters.

We estimated contemporary migration rates using bayesass v1.3 (Wilson & Rannala, 2003) using 11,000,000 MCMC iterations with the first 1,000,000 repetitions discarded as burn‐in and a sampling interval of 100 iterations. We adjusted the migration, allele frequencies, and inbreeding coefficient to 0.10, 0.35, and 0.45, respectively. Convergence was assessed using tracer v1.6 (Rambaut & Drummond, 2009).

*Mitochondrial DNA*—We randomly selected a subset of 77 individuals for mtDNA sequencing analysis from 11 populations distributed relatively evenly across the range of *A. annulatum* to maximize the amount of genetic diversity represented (Figure 1). We amplified a 900 basepair (bp) fragment consisting of the 3′ end of the cytochrome *b* gene, tRNA^Thr^, tRNA^Phe^, the intergenic space (IGS), and tRNA^Pro^ to the 5′ end of the D‐loop (control region) using the THR and DL1 primers (Shaffer & McKnight, 1996). This fragment has been found to be informative in previous phylogeographic studies of *Ambystoma* spp. (Church et al., 2003; Pauly, Bennett, Palis, & Shaffer, 2012; Pauly, Piskurek, & Shaffer, 2007; Shaffer & McKnight, 1996; Zamudio & Savage, 2003). We conducted the PCR in 25 µl volumes using 1X AmpliTaq Gold PCR Buffer (Applied Biosystems), 200 uM dNTPs, 2 mM MgCl_2_, 0.8 mM BSA, 0.4 uM of each PCR primer, 1 U AmpliTaq Gold (Life Technologies), and ~15 ng of DNA using thermocycler conditions set by Shaffer and McKnight (1996) with a 10‐min initial denaturation step at 95°C and an annealing temperature of 55°C. We bi‐directionally sequenced PCR products at the University of Missouri DNA Core Facility on an ABI 3730 DNA Analyzer (Applied Biosystems).

We edited and aligned mitochondrial sequences in geneious v8.1.9 (Kearse et al., 2012). To verify the concordance of sequence calling, we used the ClustalW method (Thompson, Higgins, & Gibson, 1994) to align raw sequence data for each individual. After verifying individual sequences, we aligned consensus sequences for all individuals using a ClustalW alignment. To guarantee equivalent comparison of haplotypes across individuals, we trimmed our alignments to a 776 bp segment of our 900 bp region which included 233 bp of the intergenic spacer, the tRNA^Pro^, and 470 bp of the D‐loop. We quantified the number of distinct haplotypes using fabox v1.41 (Villesen, 2007; accessed 11 December 2017). We visualized haplotype networks in popart v1.7 (Leigh & Bryant, 2015) using the median‐joining network method setting epsilon = 0 (Bandelt, Forster, & Rohl, 1999). Finally, we calculated haplotype diversity (*h*), nucleotide diversity (*π*), and Φ_ST_ in arlequin.

### 2.3 Demographic modeling

We inferred *A. annulatum* demographic history in the CIH using approximate Bayesian computation (ABC) implemented in diyabc v2.1.0 (Cornuet et al., 2014) using our microsatellite data. We tested a single model to estimate the divergence time between the northern and southern CIH clusters. In this model, the northern CIH and the southern CIH merge t_1_ generations before present. Across all simulations, we allowed effective population size (*N*_e_) of each cluster to vary as amphibian population sizes are highly variable in both time and space (Werner, Skelly, Relyea, & Yurewicz, 2007). We simulated 10^7^ data sets drawing from uniform prior distributions of *N*_e_ and divergence times. We calculated *A*, *H*_O_, mean allele size variation, pairwise *F*_ST_ (Weir & Cockerham, 1984), and genetic distance between populations (*δμ*)^2^ (Goldstein, Linares, Cavalli‐Sforza, & Feldman, 1995) for each simulation to compare with the observed data set in the model selection.

Our simulations assumed that microsatellites followed a generalized stepwise mutation model (Estoup, Jarne, & Cornuet, 2002) with the mean mutation rate drawn from a uniform prior distribution between 10^–5^ and 10^–3^. As suggested in the DIYABC manual, we allowed microsatellites to vary up to 40 alleles and we used default settings for all other microsatellite mutation parameters. We estimated the posterior distributions of *N*_e_ and divergence time using a local linear regression of the closest 1% of simulated data sets to the observed data following logistic transformation of parameter values (Cornuet et al., 2014, 2008). Finally, we estimated bias in the parameter estimates using DIYABC's built‐in function and simulated 500 pseudo‐observed data sets from the 90% highest posterior density distributions for each parameter. DIYABC computes the true value and compares the estimated values to the variance in simulated data sets and reports root of the relative mean integrated square error (RRMISE), average relative bias, and factor 2 score of the mode. The factor 2 score is the proportion of simulated data sets for which the estimate is at least half and at most twice the true value and values closer to 1.0 are desirable.

## 3 RESULTS

### 3.1 Microsatellite DNA

We found no individuals within the same sampling location that had a probability of being related as full siblings >95%. Similarly, we observed no sampling site or loci that deviated significantly from expectations under HWE and no loci were significantly linked. Further, we did not detect the presence of null alleles within our data set. Thus, all individuals and loci were retained for downstream analyses.

Across all populations, average *H*_O_ was 0.611 ± 0.073 (*SD*) and average *H*_E_ was 0.650 ± 0.078 (*SD*) (Table 1). Average allelic richness was 5.27 ± 1.25 (*SD*), and average rarefied allelic richness was 1.96 ± 0.89 (*SD*) across all populations (Table 1). Finally, average pairwise *F*_ST_ was 0.211 ± 0.037 (*SD*) and average *F*_IS_ was 0.058 ± 0.054 (*SD*) (Table 1).

**Table 1:** Genetic diversity for *Ambystoma annulatum* populations using 14 microsatellite loci including sample size (*N*), mean number of alleles per locus (*A*), mean rarefied allelic richness (*A*_R_), observed (*H*_O_) and expected heterozygosity (*H*_E_), and inbreeding coefficient (*F*_IS_). Cluster membership is based upon STRUCTURE assignments shown in Figure 2

Population	Abbreviation	Cluster	*N*	*A*	*A*_R_	*H*_O_	*H*_E_	*F*_IS_
St. Louis #1, MO	STL1	North	12	3.93	1.58	0.601	0.583	−0.030
St. Charles, MO	STC	North	45	4.14	1.54	0.524	0.543	0.035
St. Louis #2, MO	STL2	North	37	3.93	1.56	0.505	0.556	0.093
Warren, MO	WAR	North	41	6.36	1.69	0.685	0.693	0.011
Maries, MO	MAR	North	13	2.93	1.52	0.512	0.523	0.021
Camden, MO	CAM	North	37	6.71	1.69	0.656	0.688	0.047
Pulaski, MO	PUL	North	57	7.17	4.76	0.718	0.721	0.005
Shannon, MO	SHA	North	10	4.36	3.96	0.661	0.682	0.031
Texas, MO	TEX	North	24	4.50	1.54	0.536	0.540	0.008
Dallas, MO	DAL	North	21	6.07	1.71	0.632	0.717	0.119
Taney, MO	TAN	North	28	4.86	1.67	0.628	0.669	0.062
Stone, AR	STO	South	10	3.86	1.55	0.493	0.550	0.105
Madison, AR	MAD	South	26	6.79	1.75	0.614	0.754	0.187
Johnson, AR	JON	South	39	5.71	1.68	0.634	0.680	0.068
Franklin, AR	FRA	South	19	5.64	1.68	0.609	0.683	0.109
Cherokee, OK	CHE	South	33	5.93	1.71	0.637	0.714	0.107
Scott, AR	SCO	South	29	6.71	1.75	0.749	0.749	0.000
Global ($\bar{x}$)			28.29	5.27	1.96	0.611	0.650	0.058
North ($\bar{x}$)			29.70	5.01	2.16	0.603	0.625	0.034
South ($\bar{x}$)			26.29	5.64	1.68	0.623	0.686	0.091

**Figure 2:**
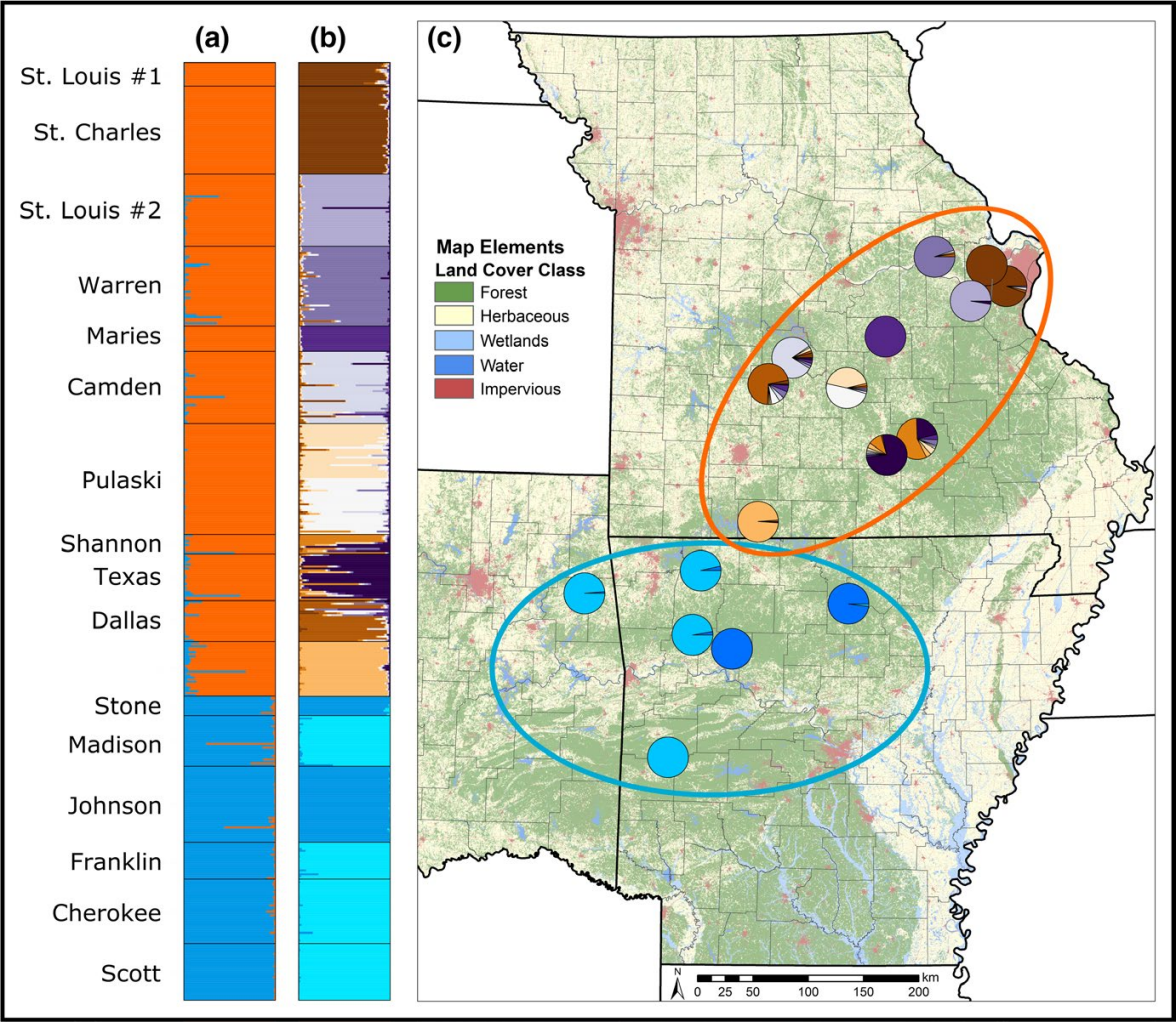
Spatial distribution of genetic cluster assignments for *Ambystoma annulatum* computed in the program structure overlaid on a simplified 2011 Land Use, Land Cover layer. Admixture coefficients for individual *A. annulatum* from (a) all sites (as identified in Table 1) and (b) from each of the two supported genetic clusters identified in our full analysis. Spatial arrangement of genetic structure for *A. annulatum* across its distribution (c). Ellipses color corresponds to the admixture assignment scores from the full analysis (a) and pie colors correspond to admixture assignment scores from the two independent analyses for hierarchical substructure of individuals within each of the two presumed genetic clusters (b)

We observed two well‐supported genetic clusters (Δ*K* = 154.49) in our structure analyses (Figure 2). The northern CIH cluster includes every Missouri sampling location and the southern CIH cluster includes all Arkansas and Oklahoma sampling sites (Figure 2). In our tests for hierarchical structure, we observed 11 well‐supported clusters within the northern cluster (Δ*K* = 81.22) and two well‐supported clusters within the southern cluster (Δ*K* = 991.54; Figure 2).

Within the northern cluster, average *H*_O_ was 0.603 ± 0.076 (*SD*) and *H*_E_ was 0.625 ± 0.082 (*SD*), whereas in the southern cluster diversity average *H*_O_ was 0.623 ± 0.075 (*SD*) and average *H*_E_ was 0.686 ± 0.069 (*SD*) (Table 1). Within the northern cluster, the average allelic richness was 5.01 ± 1.44 (*SD*) and average rarefied allelic richness was 2.16 ± 1.18 (*SD*), whereas the average allelic richness was 5.64 ± 1.03 (*SD*) and average rarefied allelic richness was 1.68 ± 0.07 (*SD*) in the southern cluster (Table 1). Finally, within the northern cluster the average *F*_ST_ was 0.191 ± 0.074 (*SD*) and *F*_IS_ was 0.034 ± 0.044 (*SD*) whereas average *F*_ST_ was 0.190 ± 0.065 (*SD*) and *F*_IS_ was 0.091 ± 0.057 (*SD*) in the southern cluster (Table 1). For all genetic diversity metrics, neither group had significantly greater diversity (all *p* > .05). The AMOVA results indicated significant subdivision among groups (and among populations within groups (Table S1).

Across the distribution of *A. annulatum*, all but one pairwise comparison of sampling locations revealed significant genetic differentiation (Table 2); the St. Louis #1 and St. Charles sampling locations in northeastern Missouri (24.6 km apart) were not significantly different (Table 2). Tests for IBD indicate that distance was a significant predictor of genetic differentiation across the entire range (Mantel's *r* = .363, *p* = .001; Figure S1); however, distance was not a significant predictor of genetic structure within each cluster (northern cluster: Mantel's *r* = .147, *p* = .196; southern cluster: Mantel's *r* = .401, *p* = .194). As in our pairwise *F*_ST_ comparisons, the highest inferred migration rates were between the St. Louis #1 and St. Charles sites in northeast Missouri; 15.1% of individuals in the St. Louis #1 site were identified as immigrants from St. Charles (Table S2). Across all sampling locations, the average percentage of migrants that assigned to their sampling site of origin per generation (i.e., the nondispersing cohort) was 84.9% (range: 67.8%–92.0%; Table S1) with the estimated average immigration rate into all other sites of 0.9% per generation (range: 0.4%–15.1%). Finally, we did not find evidence for a recent population bottleneck in terms of heterozygote excess or deficiency using a Bonferroni‐corrected *α* = 0.004 in BOTTLENECK (Table S3).

**Table 2:** Pairwise genetic and geographic distance matrix for microsatellite data for *Ambystoma annulatum*

	STL1	STC	STL2	WAR	MAR	CAM	PUL	SHA	TEX	DAL	TAN	*STO*	*MAD*	*JON*	*FRA*	*CHE*	*SCO*
STL1	–	24.6	14.1	70.3	118.5	204.1	171.2	173.4	187.0	220.0	316.3	320.6	377.6	411.1	424.2	471.9	524.5
STC	0.026	–	12.3	49.0	112.3	195.1	168.4	181.5	193.4	211.9	318.0	330.0	378.3	416.0	427.0	469.5	529.8
STL2	**0.243**	**0.296**	–	56.2	109.8	194.4	164.4	172.8	185.4	210.7	312.1	321.0	372.9	408.8	420.6	465.6	522.5
WAR	**0.163**	**0.199**	**0.228**	–	84.7	157.7	142.7	176.3	184.2	175.4	296.0	323.2	353.9	398.9	405.9	439.1	512.8
MAR	**0.292**	**0.316**	**0.339**	**0.226**	–	86.4	58.2	104.0	106.9	101.6	211.3	243.8	269.6	314.5	321.2	357.7	428.3
CAM	**0.163**	**0.206**	**0.173**	**0.146**	**0.218**	–	56.3	132.5	122.5	18.3	161.1	228.2	209.6	268.8	266.4	284.1	378.5
PUL	**0.153**	**0.196**	**0.189**	**0.077**	**0.186**	**0.113**	–	77.6	70.4	62.5	153.4	195.1	211.5	257.6	263.2	301.2	371.1
SHA	**0.211**	**0.259**	**0.183**	**0.127**	**0.285**	**0.134**	**0.105**	–	18.3	134.2	153.5	148.7	215.6	239.2	255.7	318.7	351.8
TEX	**0.300**	**0.334**	**0.219**	**0.177**	**0.352**	**0.201**	**0.168**	**0.121**	–	122.1	135.9	139.0	198.1	224.3	239.4	300.6	337.5
DAL	**0.098**	**0.155**	**0.206**	**0.084**	**0.193**	**0.100**	**0.087**	**0.146**	**0.202**	–	145.5	217.7	192.2	252.9	249.3	265.8	361.7
TAN	**0.246**	**0.290**	**0.225**	**0.141**	**0.281**	**0.169**	**0.169**	**0.089**	**0.167**	**0.157**	–	104.0	62.2	107.7	109.8	166.3	219.0
*STO*	**0.295**	**0.319**	**0.332**	**0.206**	**0.380**	**0.244**	**0.226**	**0.277**	**0.293**	**0.212**	**0.246**	–	136.9	112.8	143.8	238.9	214.3
*MAD*	**0.219**	**0.277**	**0.234**	**0.187**	**0.257**	**0.148**	**0.159**	**0.141**	**0.238**	**0.166**	**0.170**	**0.268**	–	76.1	58.5	107.1	171.4
*JON*	**0.235**	**0.279**	**0.276**	**0.195**	**0.303**	**0.187**	**0.204**	**0.222**	**0.263**	**0.161**	**0.214**	**0.232**	**0.150**	–	37.9	142.5	114.0
*FRA*	**0.274**	**0.331**	**0.270**	**0.221**	**0.314**	**0.186**	**0.198**	**0.164**	**0.264**	**0.222**	**0.191**	**0.307**	**0.094**	**0.203**	–	104.6	113.0
*CHE*	**0.249**	**0.300**	**0.212**	**0.152**	**0.283**	**0.174**	**0.182**	**0.169**	**0.235**	**0.172**	**0.195**	**0.237**	**0.116**	**0.172**	**0.157**	–	166.4
*SCO*	**0.252**	**0.301**	**0.262**	**0.204**	**0.281**	**0.197**	**0.198**	**0.210**	**0.291**	**0.198**	**0.226**	**0.282**	**0.115**	**0.213**	**0.155**	**0.148**	–

Note

Values below the diagonal represent pairwise *F*_ST_ and bold values represent comparisons that are significantly different at *p* < .001. Values above the diagonal are the pairwise great circle distances between populations (km). Population names in standard font assign to the northern CIH cluster and populations in italics assign to the southern CIH cluster (Figure 2). Shaded regions represent pairwise distance values between populations from separate genetic clusters.

### 3.2 Mitochondrial DNA

**Figure 3:**
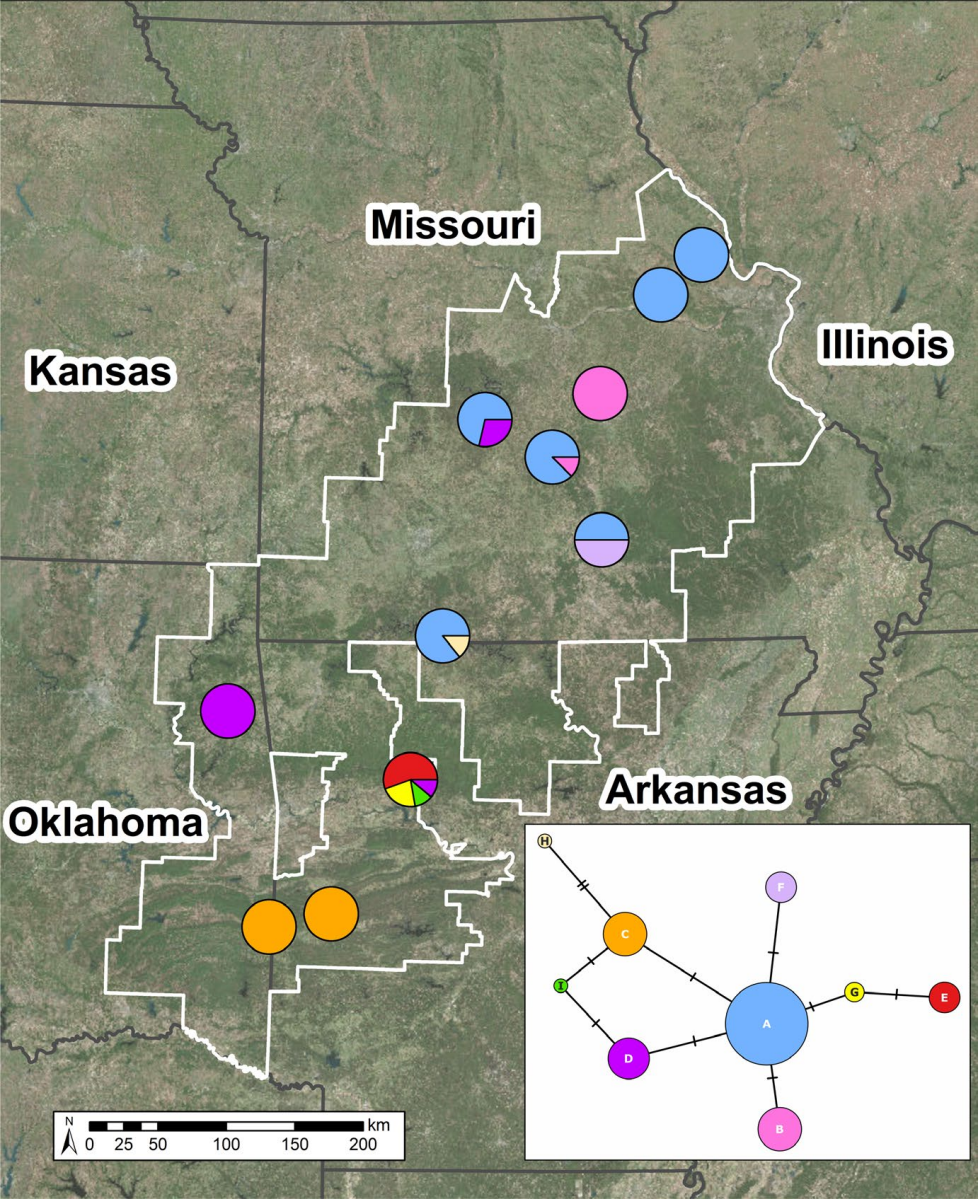
Spatial arrangement of mtDNA haplotypes by sampling location for *Ambystoma annulatum*. For each color, the proportion of the pie corresponds to the frequency of the haplotype in that population as outlined in Table 3. Colors correspond to the haplotypes represented in the median‐joining haplotype network, shown in the callout box. Each dash indicates a 1 bp difference between the connected haplotypes. White boundary corresponds to the International Union for the Conservation of Nature (IUCN) species distribution

We observed nine mtDNA haplotypes based on seven polymorphic basepairs across the 776 bp mtDNA sequence in the 77 individuals sampled (Accession No.: MN242388‐MN242396; Table 3A). The most common haplotype, haplotype A, was previously described from samples collected in southern Missouri and used as outgroups for *A. cingulatum* and *A. bishopi* phylogeographic studies (GenBank accession EU517609; Table 3A; Pauly et al., 2012). With the exception of the Maries site in northern Missouri, haplotype A was present across all sites in the northern CIH cluster (Table 3 and Figure 3). Only haplotype D was shared among the northern and southern microsatellite clusters; all other haplotypes were private to either the northern (haplotype A, B, F, H) or southern (haplotypes C, E, G, I) CIH clusters. All the Ouachita Mountain sampling locations shared a single haplotype (haplotype C; Table 3 and Figure 3). No clear patterns emerged in our median‐joining haplotype network (Figure 3) as most haplotypes were 1–3 bp diverged from haplotype A, the most common haplotype (Table 3).

**Table 3:** Summary statistics for variable nucleotide sites within our 776 bp region of *Ambystoma annulatum* mtDNA (A). Position numbers are relative to the first nucleotide of our aligned sequence. Haplotype A is the reference, and variable sites in other haplotypes are indicated with nucleotides. Dots indicate identical sequences. The number of each haplotype occurring in each sampling location and genetic cluster are indicated in columns at right. Populations are distributed among the northern CIH (OZ‐N) and southern CIH (OZ‐S and OU) microsatellite genetic clusters (Figure 2). Haplotype diversity (*h*) and nucleotide diversity (*π*) summarized by population (B)

Hap.	5	93	94	137	339	419	592	OZ‐N							OZ‐S		OU		*n*
								LIN	WAR	MAR	PUL	CAM	TEX	TAN	JON	CHE	SCO	LEF	
**(A)**																			
A	A	G	C	G	A	A	C	6	8	–	7	5	4	5	–	–	–	–	35
B	**·**	**·**	**·**	**·**	**·**	**·**	T	–	–	9	1	–	–	–	–	–	–	–	10
C	**·**	**·**	**·**	**·**	G	**·**	**·**	–	–	–	–	–	–	–	–	–	8	2	10
D	**·**	T	**·**	**·**	**·**	**·**	**·**	–	–	–	–	2	–	–	1	6	–	–	9
E	G	**·**	**·**	**·**	**·**	G	**·**	–	–	–	–	–	–	–	5	–	–	–	5
F	**·**	**·**	**·**	A	**·**	**·**	**·**	–	–	–	–	–	4	–	–	–	–	–	4
G	**·**	**·**	**·**	**·**	**·**	G	**·**	–	–	–	–	–	–	–	2	–	–	–	2
H	G	**·**	A	**·**	G	**·**	**·**	–	–	–	–	–	–	1	–	–	–	–	1
I	**·**	T	**·**	**·**	G	**·**	**·**	–	–	–	–	–	–	–	1	–	–	–	1
*n*								6	8	9	8	7	8	6	9	6	8	2	77
**(B)**																			
*h*								0.000	0.000	0.000	0.250	0.476	0.571	0.333	0.694	0.000	0.000	0.000	
*h_SD_*								0.000	0.000	0.000	0.180	0.171	0.095	0.215	0.147	0.000	0.000	0.000	
*Π*								0.000	0.000	0.000	0.000	0.001	0.001	0.001	0.002	0.000	0.000	0.000	
*π_SD_*								0.000	0.000	0.000	0.000	0.001	0.001	0.001	0.002	0.000	0.000	0.000	

Across all populations, haplotype diversity (*h*) ranged from 0.000 to 0.694 and nucleotide diversity (*π*) ranged from 0.000 to 0.002 (Table 3). Populations toward the edge of the range were fixed for a haplotype whereas populations closer to the range center had haplotype diversity between 0.250 and 0.694 (Table 3). Pairwise Φ_ST_ values based on mtDNA range from −0.040 to 1.00 (Table S4) and the average pairwise Φ_ST_ was 0.542 ± 0.370 (*SD*). Tests for IBD indicate that distance was a significant predictor of genetic differentiation based on mtDNA haplotypes (Mantel's *r* = .362; *p* = .032; Figure S1); however, distance was not a significant predictor of genetic differentiation in either cluster (northern CIH: Mantel's *r* = −.315; *p* = .950; southern CIH: Mantel's *r* = .390; *p* = .500, respectively).

### 3.3 Demographic modeling

We estimated that the divergence time between the northern and the southern CIH clusters was approximately 1,381 generations (90% HPD = 771–5,966 generations; Table 4). Given an average generation time of 2 years (Semlitsch et al., 2014), we estimated that these clusters diverged 2,762 years ago (90% HPD = 1,542–11,932 years ago). Estimated effective population sizes were lower in the northern CIH (*N*_e_ = 4,148; 90% HPD = 2,841–7,973) than in the southern CIH cluster (*N*_e_ = 8,201; 90% HPD = 4,886–15,947). Our model was well estimated as none of the simulated summary statistics were significantly different from the observed data (*p* = .135–.811).

**Table 4:** Estimates (mode and 90% highest posterior density, HPD) of the demographic parameters (scaled effective population size, *θ*; scaled divergence time, *τ*, in generations; mutation rate, *μ*) from the posterior distributions for *Ambystoma annulatum*

Parameter	Estimated values		Derived values		RRMISE	RMedAD	Avg. relative bias	Factor 2
	Mode	90% HPD	Mode	90% HPD				
*θ*_OZ‐N_	2.19 × 10^0^	1.50 × 10^–0^ to 4.21 × 10^0^	4,148	2,788–7,973	0.569	0.285	0.0451	0.960
*θ*_OZ‐S_	4.33 × 10^0^	2.58 × 10^0^ to 8.42 × 10^0^	8,201	4,886–15,947	0.380	0.195	0.3587	0.922
*τ*	7.29 × 10^–1^	4.07 × 10^–1^ to 3.15 × 10^0^	1,381	771–5,966	0.994	0.388	−0.0321	0.850
*μ*	5.28 × 10^–4^	3.66 × 10^–4^ to 9.39 × 10^–4^	–	–	0.432	0.278	−0.0588	0.918

Note

Bias estimates for the root of the relative mean integrated square error (RRMISE), average relative bias, and the factor 2 score of the mode of the posterior distribution are also included.

## 4 DISCUSSION

The patterns of *A. annulatum* genetic diversity and differentiation relate to both geologic and anthropogenic processes. Overall, we observed two strongly supported nuclear genetic clusters that correspond to a northern Ozark cluster and a southern Ozark and Ouachita Mountain cluster. Their estimated divergence time was 2,762 years before present (90% HPD = 1,542–11,932 years ago), corresponding to the early to middle Holocene (Pielou, 2008). Effective population size estimates were higher in the southern CIH cluster (*N*_e_ = 8,201) than the northern CIH cluster (*N*_e_ = 4,148). We acknowledge that the effective population sizes may be overestimated since we observed substructure within *A. annulatum*; simulation studies have shown that estimates of effective population size are higher in populations with genetic substructure than in randomly mating populations of similar size (Petit & Excoffier, 2009). For *A. annulatum* specifically, breeding census population sizes ranged from 336 to 1,971 adults at a single pond during a single breeding season (Semlitsch et al., 2014). Similarly, estimates of effective population size ranged from 2,596 to 34,310 for two congeneric species at a single sampling location (Nunziata, Lance, Scott, Lemmon, & Weisrock, 2017). Taken altogether, the higher effective population size and haplotypic diversity observed in the southern Ozarks suggests that the south‐central CIH was the center of diversity for *A. annulatum* and potential location of an ancestral population, during the Pleistocene and climatic fluctuations during the Hypsithermal intervals of the Holocene.

Further, we observed significant pairwise *F*_ST_ values between our northern and southern CIH clusters (Table 2 and Table S4) and low migration rates among populations (Table S2), which indicates more limited gene flow between sampled locations over recent time scales. When comparing the two nuclear genetic clusters with spatial patterns of genetic differentiation observed in other organisms, we found concordance in the approximate location of the *A. annulatum* phylogeographic break with those of aquatic and terrestrial fauna that exist between the northern and southern Ozarks (Hardy et al., 2002; Hutchison & Templeton, 1999; Phillips, Fenolio, Emel, & Bonett, 2017; Ray, Wood, & Simons, 2006; Routman et al., 1994). Within aquatic taxa, most studies found significant genetic differentiation between populations located in watersheds draining southward into the Arkansas River and watersheds draining northward in the Missouri or eastward into the Mississippi (Figure 2; Hardy et al., 2002; Ray et al., 2006). In some instances, such as hellbender salamanders (*Cryptobranchus alleganiensis* spp.), genetic differentiation between watersheds is pronounced enough to merit subspecific status (Crowhurst et al., 2011; Routman et al., 1994). Further, cave‐adapted species, such as grotto salamanders (*Eurycea spelaea*), show similar phylogeographic breaks between the northern and southern Ozarks that were attributed to the palaeodrainages, contemporary drainages, and subplateaus in the Ozarks (Phillips et al., 2017). Similarly, some terrestrial taxa exhibit similar patterns of genetic diversity despite these organisms not being constrained to riparian networks (Hutchison & Templeton, 1999).

We observed nine unique mtDNA haplotypes across the range of *A. annulatum* (four private to the northern CIH, four private to the southern CIH, and one shared), one of which was found within most Missouri sampling sites (Table 3 and Table S2), in comparison with the three previously observed haplotypes found in Missouri using RFLP data (Phillips et al., 2000). Since RFLP haplotypes do not provide detailed information about the underlying sequence, we cannot determine whether our haplotype A, which was found across the range of *A. annulatum* in Missouri, corresponds with the RFLP haplotype AA that Phillips et al. (2000) previously identified across the same region. Overall, we observed similar patterns of genetic differentiation in our mtDNA when compared to our microsatellite data. Most notably, Haplotype A, the most common haplotype, was only found solely within our northern CIH microsatellite cluster. Further, only one haplotype (Haplotype D) was shared among the northern and southern clusters; all other haplotypes were private to either the northern or southern CIH microsatellite cluster. In addition, all individuals from the Ouachitas were fixed for a single haplotype (Haplotype C) that was not shared with any of the Ozark sampling sites (Table 3 and Figure 3). However, Haplotype C was only 1 bp different from the most common haplotype (Haplotype A) and was intermediate to haplotypes that were found within populations in the northern CIH cluster (Figure 3).

Taken altogether, we suggest that *A. annulatum* populations experienced a bottleneck during Pleistocene glacial cycles and Hypsithermal intervals of the Holocene, with populations contracting into two refugial areas in the south‐central Ozarks and differentiated as they re‐colonized the peripheries of their contemporary range during postglacial dispersal. We observed a trend toward more haplotypes and higher genetic diversity in the center of the species distribution than toward the periphery (Table 3 and Figure 3); however, this relationship was not significant. Further, the presence of a single common haplotype across the northern CIH cluster and the estimated microsatellite cluster divergence timing supports the previously hypothesized northward expansion and lineage sorting associated with repeated contraction and recolonization of the northern Ozarks during the Holocene that coincides with the fluctuating margin of the prairie peninsula and northward movement of temperate forest habitats (Phillips et al., 2000). Further, previous studies of CIH caudates identified the Arkansas River Valley to be a phylogeographic barrier between the Ozarks and Ouachitas (Herman & Bouzat, 2016; Martin et al., 2016); however, the Arkansas River Basin was not a phylogeographic barrier for *A. annulatum* in our study. Although we surveyed a number of sites in the Ouachitas, we were only able to obtain a limited number of individuals and we were only able to obtain >10 individuals at one sampling site. Nevertheless, we observed a mtDNA haplotype private to the region and the Ouachita Mountains sampling location grouped within the southern CIH microsatellite cluster, even when testing for hierarchical substructure. This suggests that the Ouachita Mountain *A. annulation* populations may have been founded from a larger population within the south‐central Ozark Highlands.

Post‐European settlement, the CIH experienced extensive timber harvest (Cunningham, 2007; Flader, 2004), land conversion into agriculture (Jacobson, 2004; Jacobson & Primm, 1997), channelization and degradation of riparian habitats (Jacobson, 2004; Jacobson & Primm, 1997; Nigh,^2004^; Swift, 1984) and changes in the natural wildfire regime (Cutter & Guyette, 1994; Jacobson, 2004), and more recent reforestation (Flader, 2004). Despite the general records of anthropogenic disturbance in this area, there is a paucity of detail regarding the spatial and temporal scale of disturbance which precludes the ability for detailed landscape genetic analyses. However, these landscape perturbations likely decreased the amount of suitable aquatic breeding and terrestrial nonbreeding habitat available for *A. annulatum*. Thus, with decreased habitat suitability and low levels of *A. annulatum* gene flow, genetic drift in small isolated populations may have led to the observed patterns of genetic differentiation and substructure as suggested for *C. c. collaris* (Hutchison & Templeton, 1999). Although the high rates of anthropogenic disturbance in the CIH have only occurred over the last 200 years, for a species with an average generation time of 2 years, such as *A. annulatum* (Semlitsch et al., 2014), up to 100 generations may have been sufficient for the observed patterns of genetic differentiation to emerge post‐European settlement.

Worldwide, amphibian populations are experiencing declines as a result of increased rates of anthropogenic disturbance (i.e., habitat alteration, degradation, and loss), global climate change, and increased prevalence of disease (Wake & Vredenburg, 2008). Despite their designation as a species of conservation concern, *A. annulatum* populations are presumed to be relatively stable by state wildlife agencies and thus our data provide a record of the genetic diversity that is essential for evaluating the potential genetic consequences of declining populations. Although our data did not support the presence of a recent genetic bottleneck, the observed genetic substructure and differentiation present across the distribution of *A. annulatum* indicates that these populations have experienced population declines from presettlement population sizes. Further, our inability to attain sufficient sample sizes from historic localities (Figure 1) suggests that more detailed study of *A. annulatum* occupancy, abundance, and niche requirements across its distribution would provide important data for the management of this species.

## CONFLICT OF INTEREST

The authors declare no conflicts of interest associated with this work.

## AUTHOR CONTRIBUTIONS

JJB, EEP, LSE, and RDS conceived the ideas for this project; JJB, CJK, and CNS conducted the research and data analyses; LSE and RDS directed the project; and JJB led the writing of the manuscript with assistance from EEP, CJK, CNS, and LSE.

## DATA AVAILABILITY STATEMENT

All mtDNA sequences are available on GenBank (Accession No.: MN242388‐MN242396). Locality data are only provided to the county level as exact coordinates are sensitive due to the conservation status of this species; however, further information is available from the authors upon request.
