# Supplementary material for: Post‐Pleistocene differentiation in a Central Interior Highlands endemic salamander
Source: Ecol Evol. 2019 Aug 27;9(19):11171–84. doi: 10.1002/ece3.5619 (PMC6802018; doi:10.1002/ece3.5619)
Supplement: Supplementary file 1 [file ECE3-9-11171-s001.docx]

*Ecology and Evolution*

**SUPPORTING INFORMATION**

**Post-Pleistocene differentiation in a Central Interior Highlands endemic salamander**

Jacob J. Burkhart, Emily E. Puckett, Chelsey J. Beringer, Christine N. Sholy, Raymond D. Semlitsch, and Lori S. Eggert.

**Table S1:** Results of hierarchical AMOVA test in ARLEQUIN v3.5 based on nuclear microsatellite data. Population groupings defined by STRUCTURE clusters (Figure 2).

| Source of Variation | d.f. | Sum of Squares | Variance Components | Percent of Variation | *P* |
| --- | --- | --- | --- | --- | --- |
| Among Groups | 1 | 35.698 | 0.057 | 4.90 | < 0.001 |
| Among Populations within Groups | 15 | 153.264 | 0.168 | 14.36 | < 0.001 |
| Within Populations | 945 | 890.963 | 0.943 | 80.75 | < 0.001 |


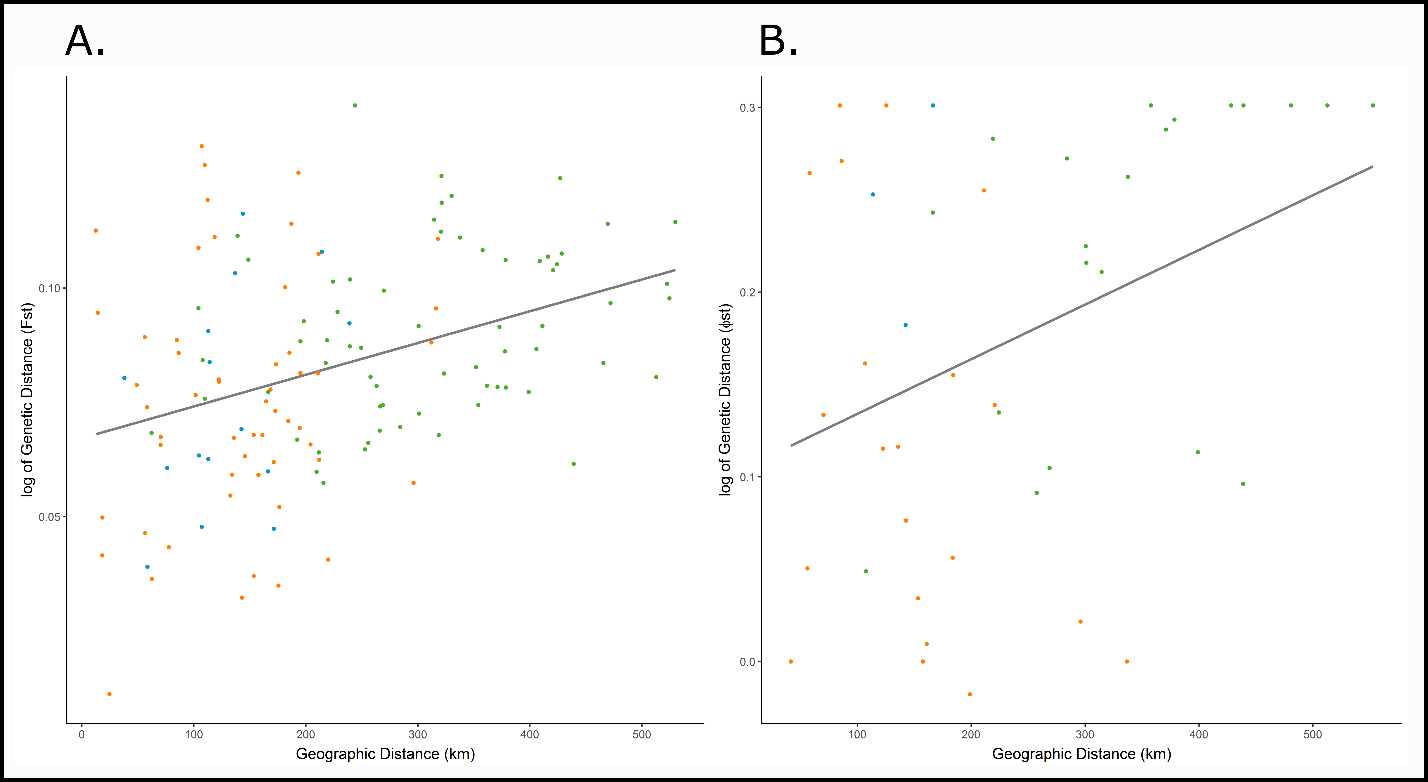


**Figure S1:** Isolation by distance (IBD) plot for all pairwise comparisons of *Ambystoma annulatum* sampling sites across the range using (A) microsatellite and (B) mtDNA markers. Tests for IBD were significant for both microsatellite (Mantel’s r = 0.367, p = 0.001) and mtDNA (Mantel’s r = 0.362, p = 0.036) data. Orange points correspond to pairwise comparisons within the northern CIH cluster, blue points correspond to pairwise comparisons among the southern CIH cluster, and green points correspond to pairwise comparisons between genetic clusters. The grey line indicates the predicted relationship between all pairwise genetic and geographic distances.

**Table S2:** Estimated *Ambystoma annulatum* contemporary migration rates. Values on the diagonal represent the percent of migrants originating in their sampled population, rows represent immigration rates, and columns represent emigration rates. Names in standard font assign to the northern cluster, names in italics to the southern cluster, and shaded regions represent estimates between populations from separate clusters.

|  | **STL1** | **STC** | **STL2** | **WAR** | **MAR** | **CAM** | **PUL** | **SHA** | **TEX** | **DAL** | **TAN** | ***STO*** | ***MAD*** | ***JON*** | ***FRA*** | ***CHE*** | ***SCO*** |
| --- | --- | --- | --- | --- | --- | --- | --- | --- | --- | --- | --- | --- | --- | --- | --- | --- | --- |
| **STL1** | **67.8±1.1** | 15.1 ± 2.9 | 1.1 ± 1.1 | 1.1 ± 1.1 | 1.2 ± 1.1 | 1.2 ± 1.1 | 1.1 ± 1.1 | 1.1 ± 1.1 | 1.1 ± 1.1 | 1.2 ± 1.1 | 1.2 ± 1.1 | 1.1 ± 1.1 | 1.1 ± 1.1 | 1.2 ± 1.1 | 1.2 ± 1.1 | 1.1 ± 1.1 | 1.2 ± 1.1 |
| **STC** | 0.5 ± 0.5 | **91.4±1.8** | 0.5 ± 0.5 | 0.5 ± 0.5 | 0.5 ± 0.5 | 0.6 ± 0.5 | 0.5 ± 0.5 | 0.5 ± 0.5 | 0.6 ± 0.6 | 0.5 ± 0.5 | 0.5 ± 0.5 | 0.5 ± 0.5 | 0.5 ± 0.5 | 0.6 ± 0.5 | 0.5 ± 0.5 | 0.5 ± 0.5 | 0.6 ± 0.5 |
| **STL2** | 0.6 ± 0.6 | 0.6 ± 0.6 | **89.9±2.1** | 0.6 ± 0.6 | 0.6 ± 0.6 | 0.6 ± 0.6 | 0.6 ± 0.6 | 0.6 ± 0.6 | 0.7 ± 0.7 | 0.6 ± 0.6 | 0.6 ± 0.6 | 0.6 ± 0.6 | 0.6 ± 0.6 | 0.6 ± 0.6 | 0.6 ± 0.6 | 0.6 ± 0.6 | 0.6 ± 0.6 |
| **WAR** | 0.6 ± 0.6 | 0.6 ± 0.6 | 0.6 ± 0.6 | **90.1±2.1** | 0.6 ± 0.6 | 0.6 ± 0.6 | 0.6 ± 0.6 | 0.6 ± 0.6 | 0.6 ± 0.6 | 1.1 ± 0.9 | 0.6 ± 0.6 | 0.6 ± 0.6 | 0.6 ± 0.6 | 0.6 ± 0.6 | 0.6 ± 0.6 | 0.6 ± 0.6 | 0.6 ± 0.6 |
| **MAR** | 1.1 ± 1.1 | 1.1 ± 1.1 | 1.1 ± 1.1 | 1.1 ± 1.1 | **82.1±3.0** | 1.1 ± 1.1 | 1.1 ± 1.1 | 1.1 ± 1.1 | 1.1 ± 1.1 | 1.2 ± 1.1 | 1.1 ± 1.1 | 1.1 ± 1.1 | 1.1 ± 1.1 | 1.1 ± 1.1 | 1.1 ± 1.1 | 1.1 ± 1.1 | 1.1 ± 1.1 |
| **CAM** | 0.6 ± 0.6 | 1.7 ± 1.2 | 0.8 ± 0.8 | 0.8 ± 0.8 | 0.8 ± 0.7 | **88.1±2.3** | 0.9 ± 0.9 | 0.6 ± 0.6 | 0.7 ± 0.7 | 0.8 ± 0.7 | 0.6 ± 0.6 | 0.6 ± 0.6 | 0.6 ± 0.6 | 0.6 ± 0.6 | 0.6 ± 0.6 | 0.6 ± 0.6 | 0.6 ± 0.6 |
| **PUL** | 0.5 ± 0.5 | 0.5 ± 0.5 | 0.5 ± 0.5 | 0.8 ± 0.7 | 0.5 ± 0.5 | 0.6 ± 0.6 | **92.0±1.7** | 0.4 ± 0.4 | 0.5 ± 0.5 | 0.5 ± 0.5 | 0.6 ± 0.6 | 0.5 ± 0.5 | 0.4 ± 0.4 | 0.4 ± 0.4 | 0.5 ± 0.5 | 0.5 ± 0.4 | 0.5 ± 0.4 |
| **SHA** | 1.3 ± 1.2 | 1.2 ± 1.2 | 1.2 ± 1.2 | 1.4 ± 1.3 | 1.2 ± 1.2 | 1.3 ± 1.2 | 1.5 ± 1.4 | **67.9±1.2** | 12.2 ± 3.1 | 1.2 ± 1.2 | 2.2 ± 1.7 | 1.2 ± 1.2 | 1.2 ± 1.2 | 1.2 ± 1.2 | 1.2 ± 1.2 | 1.2 ± 1.2 | 1.2 ± 1.2 |
| **TEX** | 0.8 ± 0.8 | 0.8 ± 0.8 | 0.9 ± 0.9 | 0.9 ± 0.9 | 0.9 ± 0.9 | 1.0 ± 1.0 | 1.1 ± 1.0 | 0.8 ± 0.8 | **85.6±2.7** | 0.9 ± 0.8 | 1.1 ± 1.0 | 0.8 ± 0.8 | 0.8 ± 0.8 | 0.8 ± 0.8 | 0.8 ± 0.8 | 1.1 ± 1.0 | 0.8 ± 0.8 |
| **DAL** | 0.9 ± 0.8 | 1.2 ± 1.1 | 1.0 ± 1.0 | 2.1 ± 1.4 | 1.2 ± 1.1 | 1.2 ± 1.2 | 1.1 ± 1.0 | 0.9 ± 0.9 | 0.9 ± 0.9 | **83.2±3.0** | 0.9 ± 0.9 | 0.9 ± 0.8 | 1.0 ± 0.9 | 1.0 ± 1.0 | 0.9 ± 0.9 | 0.9 ± 0.9 | 0.9 ± 0.9 |
| **TAN** | 0.7 ± 0.7 | 0.8 ± 0.7 | 0.7 ± 0.7 | 0.7 ± 0.7 | 0.8 ± 0.7 | 0.8 ± 0.8 | 0.7 ± 0.7 | 0.7 ± 0.7 | 0.8 ± 0.8 | 0.7 ± 0.7 | **88.0±2.4** | 0.7 ± 0.7 | 0.8 ± 0.8 | 0.8 ± 0.7 | 0.8 ± 0.7 | 0.7 ± 0.7 | 0.7 ± 0.7 |
| ***STO*** | 1.2 ± 1.2 | 1.3 ± 1.2 | 1.3 ± 1.3 | 1.2 ± 1.2 | 1.2 ± 1.2 | 1.3 ± 1.3 | 1.3 ± 1.2 | 1.2 ± 1.2 | 1.3 ± 1.3 | 1.2 ± 1.2 | 1.3 ± 1.2 | **80.1±3.1** | 1.2 ± 1.2 | 1.3 ± 1.2 | 1.2 ± 1.2 | 1.2 ± 1.2 | 1.2 ± 1.2 |
| ***MAD*** | 0.8 ± 0.8 | 0.8 ± 0.8 | 0.8 ± 0.8 | 0.9 ± 0.8 | 0.8 ± 0.8 | 1.3 ± 1.1 | 0.8 ± 0.8 | 0.8 ± 0.8 | 1.1 ± 1.0 | 1.1 ± 1.0 | 0.9 ± 0.8 | 0.8 ± 0.8 | **85.8±2.6** | 0.8 ± 0.8 | 1.0 ± 1.0 | 1.0 ± 0.9 | 0.8 ± 0.8 |
| ***JON*** | 0.6 ± 0.6 | 0.6 ± 0.6 | 0.6 ± 0.6 | 0.6 ± 0.6 | 0.6 ± 0.6 | 0.6 ± 0.6 | 0.6 ± 0.6 | 0.6 ± 0.6 | 0.6 ± 0.6 | 0.6 ± 0.6 | 0.6 ± 0.6 | 0.6 ± 0.6 | 0.6 ± 0.6 | **90.5±2.0** | 0.6 ± 0.6 | 0.6 ± 0.6 | 0.6 ± 0.6 |
| ***FRA*** | 0.9 ± 0.9 | 0.9 ± 0.9 | 0.9 ± 0.9 | 0.9 ± 0.9 | 0.9 ± 0.9 | 0.9 ± 0.9 | 0.9 ± 0.9 | 0.9 ± 0.9 | 0.9 ± 0.9 | 1.0 ± 0.9 | 0.9 ± 0.9 | 1.0 ± 0.9 | 1.2 ± 1.1 | 0.9 ± 0.9 | **84.9±2.8** | 1 .0 ± 1.0 | 0.9 ± 0.9 |
| ***CHE*** | 0.7 ± 0.7 | 0.7 ± 0.6 | 0.8 ± 0.7 | 0.7 ± 0.7 | 0.7 ± 0.7 | 0.7 ± 0.7 | 0.7 ± 0.7 | 0.7 ± 0.7 | 0.7 ± 0.7 | 0.7 ± 0.7 | 0.7 ± 0.7 | 0.7 ± 0.7 | 0.7 ± 0.7 | 0.7 ± 0.7 | 0.7 ± 0.6 | **89.2±2.2** | 0.6 ± 0.6 |
| ***SCO*** | 0.8 ± 0.8 | 0.7 ± 0.7 | 0.8 ± 0.7 | 0.7 ± 0.7 | 0.7 ± 0.7 | 0.7 ± 0.7 | 0.7 ± 0.7 | 0.7 ± 0.7 | 0.8 ± 0.7 | 0.7 ± 0.7 | 0.7 ± 0.7 | 0.7 ± 0.7 | 0.7 ± 0.7 | 0.7 ± 0.7 | 0.7 ± 0.7 | 0.8 ± 0.8 | **88.3±2.3** |

**Table S3:** Mean expected heterozygosity and Bonferroni corrected significance values for heterozygote excess or deficiency using the two-phase mutational model (TPM) with 5% multistep mutations in the program Bottleneck. Bold values indicate significant heterozygote excess or deficiency at *P* < 0.004 and italics values indicate significance at *P* < 0.05. We did not find consistent patterns of heterozygote excess or deficiency in any sampling location.

| **Population** | **Cluster** | **Excess** | **Deficiency** | **Mean H_E_** |
| --- | --- | --- | --- | --- |
| St. Louis #1, MO | North | 0.476 | 0.548 | 0.584 |
| St. Charles | North | 0.665 | 0.357 | 0.543 |
| St. Louis #2, MO | North | 0.073 | 0.936 | 0.556 |
| Warren, MO | North | 0.313 | 0.709 | 0.693 |
| Maries, MO | North | *0.039* | 0.966 | 0.522 |
| Camden, MO | North | 0.903 | 0.108 | 0.688 |
| Pulaski, MO | North | 0.076 | 0.935 | 0.618 |
| Shannon, MO | North | 0.208 | 0.806 | 0.667 |
| Texas, MO | North | 0.768 | 0.251 | 0.540 |
| Dallas, MO | North | 0.380 | 0.643 | 0.715 |
| Taney, MO | North | 0.097 | 0.914 | 0.668 |
| Stone, AR | South | 0.706 | 0.318 | 0.548 |
| Madison, AR | South | 0.292 | 0.729 | 0.751 |
| Johnson, AR | South | 0.313 | 0.709 | 0.679 |
| Franklin, AR | South | 0.271 | 0.749 | 0.681 |
| Cherokee, OK | South | 0.077 | 0.932 | 0.712 |
| Scott, AR | South | 0.163 | 0.852 | 0.749 |

**Table S4:** Pairwise genetic and geographic distance matrix for *A. annulatum* mtDNA haplotypes. Values below the diagonal represent pairwise Φ_ST_ with values in and values above the diagonal are the pairwise great circle distances (km). Populations names in standard font assign to the northern cluster and populations in italic font assign to the southern cluster (Figure 2). The “OU” sampling site includes both the Scott County, AR and LeFlore County, OK sampling locations. Shaded regions represent pairwise distance values between populations from separate genetic clusters. Bolded values represent pairwise comparisons that are significantly different at *p* < 0.05 and values in italics are trending towards significance at *p* < 0.10.

|  | LIN | WAR | MAR | PUL | CAM | TEX | *TAN* | *JON* | *CHE* | *OU* |
| --- | --- | --- | --- | --- | --- | --- | --- | --- | --- | --- |
| LIN | – | 41.68 | 125.57 | 198.95 | 183.74 | 220.71 | 336.80 | 438.72 | 480.77 | 552.68 |
| WAR | 0.000 | – | 84.72 | 157.68 | 142.73 | 184.23 | 296.04 | 398.93 | 439.09 | 512.78 |
| MAR | **1.000** | **1.000** | – | 86.39 | 58.17 | 106.91 | 211.34 | 314.51 | 357.73 | 428.26 |
| PUL | -0.040 | 0.000 | **0.866** | – | 56.34 | 122.50 | 161.13 | 268.80 | 284.11 | 378.46 |
| CAM | 0.138 | 0.192 | **0.838** | 0.123 | – | 70.44 | 153.37 | 257.62 | 301.20 | 371.05 |
| TEX | *0.377* | *0.429* | **0.450** | *0.304* | **0.360** | – | 135.88 | 224.27 | 300.59 | 337.52 |
| *TAN* | 0.000 | 0.051 | **0.799** | 0.022 | 0.082 | *0.307* | – | 107.69 | 166.30 | 218.97 |
| *JON* | **0.248** | **0.298** | **0.625** | **0.273** | **0.234** | **0.364** | 0.119 | – | 142.54 | 113.99 |
| *CHE* | **1.000** | **1.000** | **1.000** | **0.872** | **0.644** | **0.678** | **0.750** | **0.521** | – | 166.35 |
| *OU* | **1.000** | **1.000** | **1.000** | **0.965** | **0.941** | **0.830** | **0.919** | **0.790** | **1.000** | – |
